# Prevalence and prognostic implications of psychological distress in patients with gastric cancer

**DOI:** 10.1186/s12885-017-3260-2

**Published:** 2017-04-20

**Authors:** Gun Min Kim, Seung Jun Kim, Su Kyung Song, Hye Ryun Kim, Beo Deul Kang, Sung Hoon Noh, Hyun Cheol Chung, Kyung Ran Kim, Sun Young Rha

**Affiliations:** 1 Division of Medical Oncology, Department of Internal Medicine, Yonsei University College of Medicine, 50 Yonsei-ro, Seodaemun-gu, Seoul, 120-752 South Korea; 2 Department of Psychiatry, Konyang University College of Medicine, Daejeon, South Korea; 3 Outpatient Clinic, Department of Medical Oncology, Severance Hospital, Seoul, South Korea; 4 Division of Hematology and Medical Oncology, Department of Internal Medicine, Seoul National University Bundang Hospital, Seongnam, South Korea; 5 Department of Surgery, Yonsei University College of Medicine, Seoul, South Korea; 6 Department of Psychiatry, Yonsei University College of Medicine, Seoul, South Korea

**Keywords:** Psychological distress, Gastric cancer, Prognosis, Survival

## Abstract

**Background:**

The aim of this study was to investigate the prevalence and prognostic significance of psychological distress in gastric cancer patients.

**Methods:**

The study population included 229 gastric cancer patients visiting Yonsei Cancer Center between November 2009 and March 2011. The distress was measured by available tools including the Modified Distress Thermometer (MDT), Hospital Anxiety and Depression Scale (HADS), and Center for Epidemiologic Studies–Depression Scale (CES-D). Patients with psychological distress were defined as those who scored above the cut-off values in both the MDT and either one of the HADS or CES-D.

**Results:**

The median age of patients was 56 (range, 20 to 86) and 97 (42.4%) patients were with stage IV disease status at enrollment. The overall prevalence of psychological distress was 33.6% (95% CI: 27.5–39.8%) in 229 gastric cancer patients. In multiple logistic regression analysis, lower education level (odds ratio [OR] 2.39; 95% confidence interval [CI] 1.11–5.17, *P* = 0.026) and higher disease stage (OR 2.72; 95% CI 1.47–5.03, *P* = 0.001) were associated with psychological distress. In stage I-III disease, patients with psychological distress had worse disease-free survival (DFS) (5-year DFS rate: 60% vs 76%, *P* = 0.49) compared with those without psychological distress. In stage IV disease (*n* = 97), patients with psychological distress showed poorer overall survival than those without psychological distress (median OS (Overall Survival): 12.2 vs. 13.8 months, *P* = 0.019).

**Conclusion:**

Psychological distress is common in patients with all stages of gastric cancer and is associated with worse outcomes.

**Electronic supplementary material:**

The online version of this article (doi:10.1186/s12885-017-3260-2) contains supplementary material, which is available to authorized users.

## Background

Cancer diagnosis and treatment is a significantly stressful event that generates psychological distress in a large number of cancer patients. Psychological distress is generally defined as a state of emotional suffering characterized by symptoms of depression and anxiety [1]. Approximately 20–40% of cancer patients show a significant level of psychological distress [2, 3]. Distress can exert a negative impact on their treatment adherence, quality of life (QOL), pain, and even on survival [4–8]. Recently, in most solid cancers including gastric cancer, the multidisciplinary approach is becoming more important for the decision of cancer treatment strategy and psychosocial support is one of the essential parts of the multidisciplinary approach [9, 10].

Gastric cancer is the second leading cause of cancer deaths in the world [11]. Most patients with gastric cancer have advanced to an incurable stage at the time of diagnosis, which induces tremendous psychological stress. Even if the patient is diagnosed with early-stage gastric cancer, they suffer from not only the diagnosis of cancer but also surgery itself [12]. Therefore, the importance of psychological distress in gastric cancer patients for the decision of treatment will continue to grow.

Few studies have reported on the prevalence or the nature of psychological distress in gastric cancer patients. Tavoli et al. reported that overall 47.2% and 57% of patients with gastrointestinal cancer scored high on both anxiety and depression respectively [13]. We previously reported the prevalence and associated factors of psychological distress in all types of cancer patients and found that 28.3% of gastric cancer patients had psychological distress [14]. Further, there is no report of the survival impact of psychological distress in gastric cancer patients.

The aim of this study was to evaluate the prevalence and prognostic impact of psychological distress in gastric cancer patients.

## Methods

### Study population

Patients were eligible for study participation if they met the following criteria: 1) histologically confirmed gastric adenocarcinoma; 2) age of >20 years; 3) Eastern Cooperative Oncology Group (ECOG) performance status 0–3; 4) the ability to read and understand the questionnaire; 5) the ability to communicate in written and spoken language; and 6) willing and able to provide written informed consent.

Patients with operable gastric cancer underwent surgery and then received adjuvant chemotherapy according to the final pathologic stage. Stage IV gastric cancer patients received a standard treatment of palliative systemic chemotherapy with or without palliative gastrectomy.

**Fig. 1:**
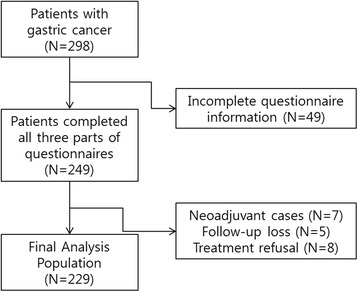
Study Population

Patients with gastric cancer visiting Yonsei Cancer Center, Severance Hospital in Seoul, Korea between November 2009 and March 2011 were included. Patients were enrolled at the first visit to the medical oncology department. During the study period, 298 gastric cancer patients agreed to complete questionnaires for screening distress. Among them, 249 (83.5%) patients completed all questionnaires. We excluded 20 patients in the final analysis due to several reasons (such as neoadjuvant chemotherapy, follow-up loss, treatment refusal, etc.) Finally, we analyzed the data of 229 gastric cancer patients (Fig. 1).

### Study procedure

An Oncology Certified Nurse (OCN) explained the purpose and procedure of the distress screening program to gastric cancer patients in the oncology outpatient clinic. Patients who signed informed consent forms completed questionnaires containing the Modified Distress Thermometer (MDT), Hospital Anxiety and Depression Scale (HADS), and Center for Epidemiologic Studies-Depression Scale (CES-D), and questions about socio-demographic and clinical status. Cancer-related information including cancer stage, disease-free survival, overall survival, and adjuvant chemotherapy were collected in electronic medical records. The patients who were identified as harboring psychological distress were referred to a psychiatrist for further evaluation and treatment for psychological distress by a medical oncologist. An independent psychiatric doctor reviewed the data without any clinical information and informed the oncologist about the status of psychological distress.

### Measure of psychological distress

The following three self-administered questionnaires were used to evaluate the psychological distress of enrolled patients.

The MDT is an easily used screening tool to measure the severity of psychological symptoms including anxiety, insomnia, and depression, and the degree of functional impairment due to these symptoms. It contains three subscales: MDT-anxiety, MDT-insomnia, and MDT-depression. Patients are required to circle the number that corresponds to their severity of distress and the degree of functional impairment on an 11-point visual analogue scale ranging from 0 to 10 by referring to the previous week. More than 4 points in both the severity and impairment scales in each distress symptom indicates that it is necessary to refer to a psychiatrist [15].

The HADS is widely used to measure anxiety and depression in patients with medical illness. It is considered as an effective screening tool to evaluate psychological distress in cancer patients [16, 17]. It consists of two subscales (HADS-A and HADS-D) that evaluate anxiety and depression, respectively. We used the Korean-validated version of the HADS for this study. Scores for each subscale range from 0 to 21 and a cut-off score of 8 was used, which had been previously reported to show good sensitivity and specificity (89.2 and 82.5%, respectively) in a Korean population [18]. Patients who scored above the cut-off score in either the HADS-A or HADS-D scale were defined as one with psychological distress based on the HADS.

The CES-D is a 20-item tool used to evaluate depressive symptoms in the general population. The Korean version of the CES-D was used in this study and we defined a CES-D score of 21 as the cut-off score, which was reported to be the threshold for the purpose of estimating the prevalence of depressive symptoms in Korean patients [19].

In this study, patients with psychological distress were defined as those who scored above the cut-off value in both MDT scales and either one of the HADS or CES-D scales.

### Statistical analysis

In order to compare psychological distress with regard to socio-demographic and clinical characteristics, chi-square tests were conducted for categorical and nominal variables, and independent samples t-test and analyses of variance (ANOVA) were performed for continuous variables. Standard univariate descriptive statistics were used to calculate the prevalence of distress. Logistic regression analysis was conducted to evaluate the factors that show the strongest association with psychological distress. Univariate and multivariate Cox proportional hazards models were used to determine the effect of independent predictors on survival times. Disease-free survival (DFS) was defined as the time from the date of surgery to the disease recurrence or death from any other causes. All statistical analysis was conducted using SPSS, software version 23.0 (SPSS Inc.).

## Results

### Patient characteristics

Table 1 shows the socio-demographic and clinical data of the evaluable 229 patients. The median age of the patients was 56 years (range: 20–86), and 167/229 (73%) were male. Most patients were married (196/229 [85.6%]), and more than half of the participants were high school educated or higher (178/229 [77.8%]) and unemployed (121/229 [52.8%]). Patients with metastatic or recurrent disease (stage IV) at enrollment were 42.4%. Most of the patients were non-smokers (79.9%) and physically active (ECOG 0 or 1: 94.7%). Among stage I-III disease patients (*n* = 132), 83 patients received adjuvant chemotherapy after surgery. The proportion of patients who received adjuvant chemotherapy in stage I, II, and III was 2.9%, 81.8%, and 83.1%, respectively. The most commonly used chemotherapy regimen was platinum-based doublet, for 67.5% with an adjuvant aim and 73.1% with a palliative aim. Of 97 patients with recurrent or metastatic disease at enrollment, more than half of the patients (53.6%) had peritoneal metastases at diagnosis and 84.5% received palliative chemotherapy in the first-line setting. Fifteen patients with recurrent or metastatic disease underwent palliative surgery for several purposes such as clinical trials (*n* = 4), good responder to palliative chemotherapy (*n* = 8), and palliation of symptoms (*n* = 3).

**Table 1:** Baseline characteristics

	*N* = 229	%
Age		
Median	56	
Range	20–86	
Gender		
Male	167	72.9
Female	62	27.1
ECOG Performance		
0	121	52.8
1	96	41.9
2–3	12	5.2
Smoking		
Smoker	46	20.1
Non-Smoker	183	79.9
Marital status		
Married	196	85.6
Single	17	7.4
Widowed	12	5.2
Divorced	4	1.7
Educational level		
Elementary school	24	10.5
Middle school	27	11.8
High school	86	37.6
Undergraduate	74	32.3
Graduate school	18	7.9
Employment status		
Full-time job	82	35.8
Part-time job	26	11.4
Unemployed	82	35.8
Housewife/Student	39	17
Histology		
Tubular adenocarcinoma	161	70.3
Signet ring cell carcinoma	58	25.3
Mucinous carcinoma	5	2.2
Others	5	2.2
Adjuvant chemotherapy		
Platinum-based doublet (SP or FP)	56/83	67.5
TS-1 monotherapy	22/83	26.5
Others	5/83	6
Initial metastasis site		
Peritoneum	52/97	53.6
Distant LN	41/97	42.3
Liver	28/97	28.9
Bone	12/97	12.4
Lung	9/97	9.3
Brain	2/97	2.1
Others	19/97	19.6
Palliative chemotherapy		
No	15/97	15.5
Yes	82/97	84.5
Palliative chemotherapy regimen		
Platinum-based doublet (SP or FP)	60/82	73.2
Taxane-based regimen	12/82	14.6
Triplet (DCF)	2/82	2.4
Others	8/82	9.7
AJCC stage at enrollment		
1	34	14.8
2	33	14.4
3	65	28.4
4	97	42.4

### Prevalence of psychological distress in gastric cancer patients

**Fig. 2:**
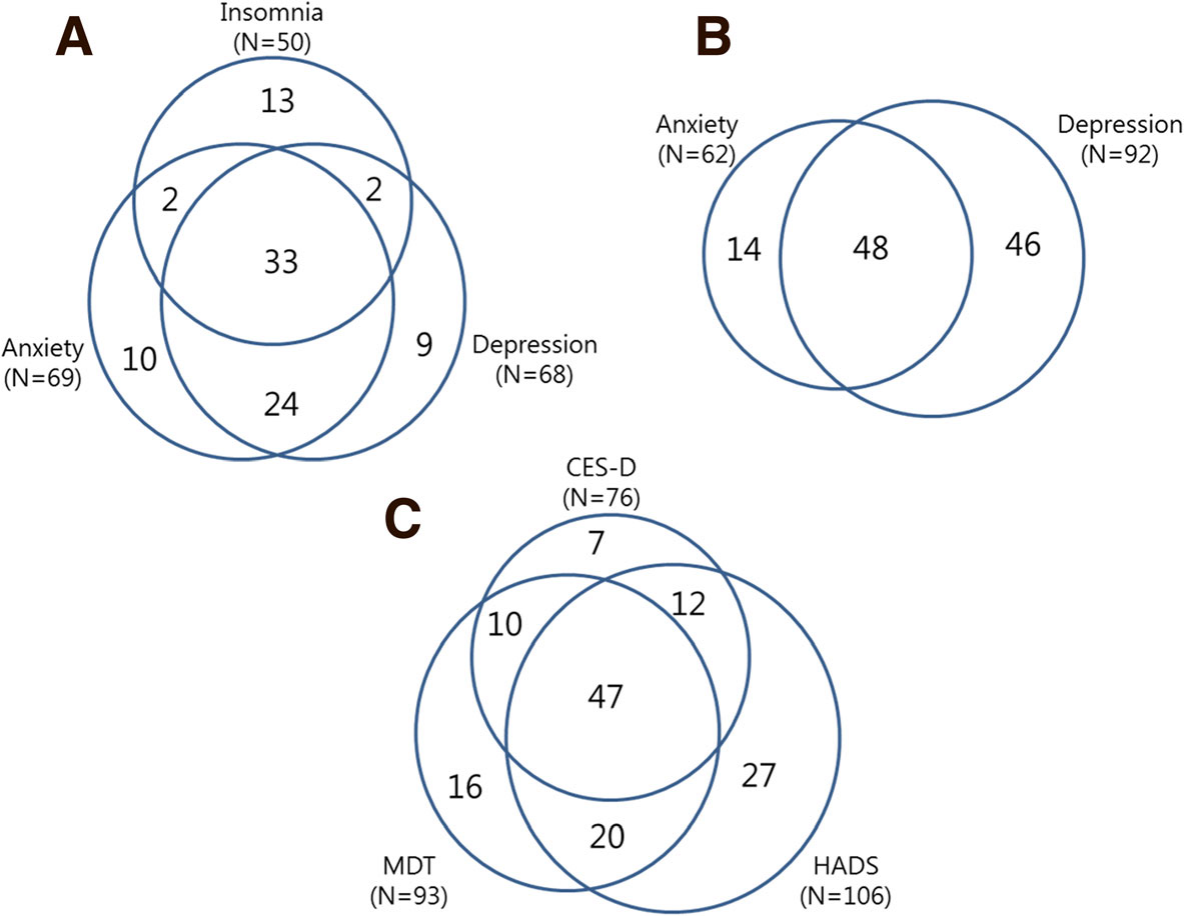
Concordance rate between distress measure parameters (**a**) MDT (**b**) HADS (**c**). All three distress parameters; Shaded area are the patient who was defined as psychological distress in this study

The results of distress screening through the questionnaires are shown in Table 2. Among the 229 patients, 77 (33.6%) were identified as patients with psychological distress. Using the MDT, 50 patients reported insomnia (21.8%), 69 anxiety (30.1%), or 68 depression (29.7%). The number of patients who scored above the cutoff value in HADS-A, HADS-D, and CES-D was 62 (27.1%), 92 (40.2%), and 76 (33.2%), respectively. Concordance between the parameters are displayed in Fig. 2. Among 77 patients with psychological distress, 61% had positive results for all three methods. Patients who are in the shaded area in Fig. 2c were defined as patients with psychological distress. The prevalence of psychiatric illness diagnosed by the psychiatrist is shown in Additional file 1: Table S1.

**Table 2:** Prevalence of psychological distress by disease stage

	All Patients		Stage I-III		Stage IV		
	*N* = 229	%	*N* = 132	%	*N* = 97	%	*P-value*
MDT	93	40.6	46	34.8	47	48.5	0.038
Insomnia	50	21.8	28	21.2	22	22.7	0.79
Anxiety	69	30.1	30	22.7	39	40.2	0.004
Depression	68	29.7	31	23.5	37	38.1	0.016
HADS	106	46.3	52	39.4	54	55.7	0.015
HADS-A	62	27.1	29	22	33	34	0.043
HADS-D	92	40.2	45	34.1	47	48.5	0.028
CES-D	76	33.2	38	28.8	38	39.2	0.099
Psychological distress	77	33.6	35	26.5	42	43.3	0.008

*MDT* Modified Distress Thermometer, *HADS* Hospital Anxiety and Depression Scale, *CES-D* Center for Epidemiologic Studies-Depression Scale

### Risk factors of psychological distress

Table 3 describes the comparison of socio-demographic and clinical factors between patients with and without psychological distress. Patients with psychological distress were significantly higher in females (*P* = 0.024), the unemployed (*P* = 0.02), those with lower educational background (*P* = 0.021), and those at an advanced stage (*P* = 0.008). The logistic regression analysis showed that education, disease stage, and smoking status maintained a statistically significant association with psychological distress (Additional file 1: Table S2). The patients with low education levels were 2.39 times (95% CI, 1.11–5.17, *P* = 0.026) more likely to have psychological distress than those with high education levels. The patients with stage IV incurable disease stage were 2.72 times (95% CI, 1.47–5.03, *P* = 0.001) more likely to have psychological distress than those with a curable disease stage.

**Table 3:** Comparison of socio-demographic characteristics between the patients with psychological distress and those without psychological distress

	Patients without psychological distress		Patients with psychological distress		
	*N* = 152	%	*N* = 77	%	*P-value*
Age					0.289
Mean (SD)	57 (13)		55 (12.7)		
Gender					0.024
Male	118	77.6	49	63.6	
Female	34	22.4	28	36.4	
ECOG Performance					0.055
0	82	53.9	39	50.6	
1	66	43.4	30	39	
2–3	4	2.6	8	10.4	
Smoking					0.056
Smoker	116	76.3	67	87	
Non-Smoker	36	23.7	10	13	
Marital status					0.247
Married	133	87.5	63	81.8	
Unmarried	19	12.5	14	18.2	
Educational status					0.021
≤ Middle school	27	17.8	24	31.2	
≥ High school	125	82.2	53	68.8	
Employment status					0.02
Employed	80	52.6	28	36.4	
Unemployed	72	47.4	9	63.6	
Disease stage at enrollment					0.008
1–3	97	63.8	35	45.5	
4	55	36.2	42	54.5	
Disease status at enrollment					0.072
Post-op status	64	42.1	23	29.9	
Pre-op or metastatic	88	57.9	54	70.1	

*SD* Standard Deviation, *ECOG* Eastern Cooperative Oncology Group

### Survival analysis

**Fig. 3:**
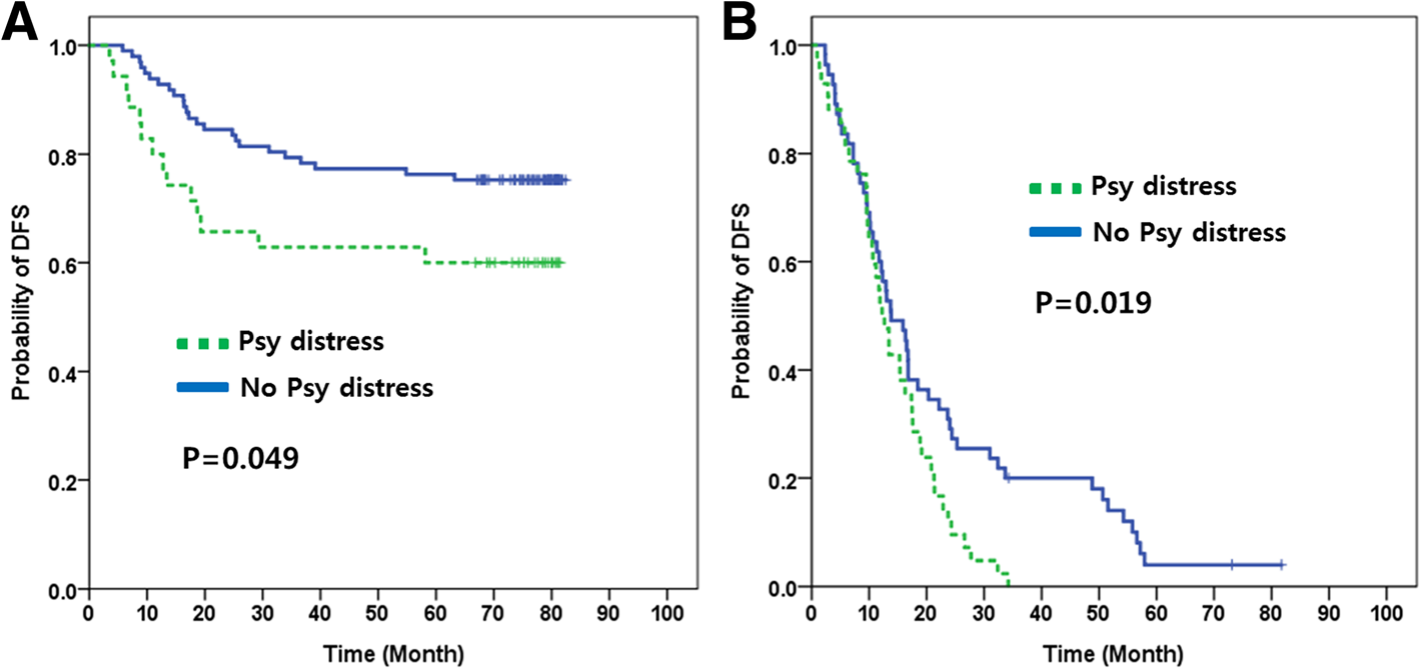
(**a**) DFS in Stage I-III disease and (**b**) OS in Stage IV disease by psychological distress

The median follow-up duration of the entire cohort was 42.5 months. We analyzed survival data divided into two subsets (curable disease, stage I-III vs. incurable disease, stage IV). DFS by TNM (Tumor/Node/Metastasis) sub-stage are shown in Additional file 1: Figure S1. In stage I-III disease, patients who have psychological distress had worse disease-free survival (5-year DFS rate: 60% vs 76%, *P* = 0.49, Fig. 3a). In stage IV disease, patients with psychological distress had worse OS than those without psychological distress (median OS: 12.2 vs. 13.8 months, *P* = 0.019, Fig. 3b).

The Cox multivariate analysis model including age, gender, ECOG, adjuvant chemotherapy, marriage, education, employment, and psychological distress in stage III disease showed that adjuvant chemotherapy (Hazzard ratio [HR] 7.23, 95% CI 2.27–22.95, *P* < 0.001) and psychological distress (HR 2.47, 95% CI 1.07–5.68, *P* = 0.034) were associated with shorter disease free survival (Table 4).

**Table 4:** Disease-free survival multivariate analysis in Stage III Disease

		Multivariate analysis		
Factor	N	Hazzard ratio	95% CI	*p*-value
Gender				
Male	39	1		
Female	26	2.05	0.85–4.91	0.108
Age				
< 65	32	1		
≥ 65	33	1.34	0.47–3.79	0.585
ECOG Performance				
0	28	1		
> 1	37	1.27	0.54–2.97	0.584
Adjuvant chemotherapy				
Yes	54	1		
No	11	7.23	2.27–22.95	<0.001
Psychological distress				
No	45	1		
Yes	20	2.47	1.07–5.68	0.034
Marital status				
Married	54	1		
Unmarried	11	1.562	0.53–4.59	0.417
Educational status				
≤ Middle school	18	1		
≥ High school	47	0.49	0.21–1.39	0.154
Employment status				
Employed	29	1		
Unemployed	36	1.98	0.72–5.45	0.187

## Discussion

Psychological support is an important part of the multidisciplinary approach, but there is no study that specifically evaluated the psychological distress in gastric cancer, which is the most common cancer in Korea. To our knowledge, this is the first study to explore the prevalence and prognostic impact of psychological distress among a large number of patients with gastric cancer. In our study cohort of gastric cancer patients, significant psychological distress was identified in 33.6% of patients. In addition, we found that psychological distress has a poor prognostic impact for gastric cancer patients.

The presence of psychological distress is a risk factor for treatment noncompliance. A meta-analysis showed that noncompliance was greater in patients with depression compared to non-depressed patients [20]. Therefore, it is important to identify the patients who may be vulnerable to psychological distress to improve treatment adherence. We found that the patients with advanced disease, low levels of education, and who were female were found to be significantly vulnerable to psychological distress. These findings are comparable to previous studies [21–24]. Several studies reported a higher prevalence of psychological distress in patients with lower education. Lower coping skills seem to contribute to the higher rate of psychological distress in those with little education [24].

Kuchler et al. reported that patients with gastrointestinal cancer including stomach, pancreatic, liver, or colorectal cancer who received a formal program of psychotherapeutic support during their hospital stay showed a better survival than those who did not [25]. In this study population, there were few recurrence cases in stage I or II disease; therefore, we performed a multivariate survival analysis in stage III disease. Although not statistically significant, patients who had psychological distress were less likely to receive adjuvant chemotherapy than those who did not. Treatment non-compliance related with adjuvant therapy could be one of the reasons for the poor survival in patients with psychological distress.

There are many screening tools with variable formats and lengths for evaluating psychological distress. However, it is not clear which screening method is appropriate for cancer patients. The Distress Thermometer (DT) is widely used due to its simplicity, but it is generally poor accuracy was pointed as a limitation [26]. To compensate this weakness, we added two other scales for the evaluation of psychological distress. In this study, we also have to consider the balance between minimizing the burden on patients and maximizing validity of data. We selected three short-length screening tools—MDT, HADS, and CES-D—that were validated by several studies [27]. We also previously reported the sensitivity and specificity of these tools in Korean cancer patients [14]. However, considering that 49 (16.5%) patients did not answer the questions completely, filling out all three questionnaires was clearly some burden to cancer patients. Further studies are warranted to develop efficient tools reflecting the distinct characteristics of Korean culture.

There are several limitations in our study. First, the timing of surveillance was not consistent in all populations. Some patients filled out the questionnaires after hearing the prognosis of their disease, and others did when not knowing their prognosis or treatment plan. Second, we could not follow the development of psychological distress over the trajectory of cancer because of our cross-sectional design. Third, these data were obtained by self-report, and it is possible that patients may have under- or overestimated their status. Fourth, systemic chemotherapy for metastatic gastric cancer was not standardized.

Despite these limitations, this study has important strengths. First, our study demonstrates that approximately one-third of gastric cancer patients have significant psychological distress, especially in low-educated patients with advanced stage disease. The patients with psychological distress showed poor survival outcomes that may be related with treatment non-compliance.

## Conclusions

Psychological distress is common in patients with all stages of gastric cancer and is associated with worse outcomes. From these results, we conclude that we need to pay attention to the psychological status of gastric cancer patients. Ultimately, further research is needed to investigate whether psychotherapeutic interventions would decrease the distress and improve survival outcomes in gastric cancer patients.

## Additional file

Additional file 1: Supplementary figure and tables. Disease free survival differences by TNM sub-stage, psychiatric illness by age group. (DOC 109 kb)

## Abbreviations

ANOVA: Analyses of variance
CES-D: Center for Epidemiologic Studies–Depression Scale
CI: Confidence interval
DFS: Disease-free survival
DT: Distress Thermometer
ECOG: Eastern Cooperative Oncology Group
HADS: Hospital Anxiety and Depression Scale
HR: Hazzard ratio
MDT: Modified Distress Thermometer
OCN: Oncology Certified Nurse
OR: Odds ratio
OS: Overall Survival
QOL: Quality of life
TNM: Tumor/Node/Metastasis
