# Supplementary material for: Prevalence and prognostic implications of psychological distress in patients with gastric cancer
Source: BMC Cancer. 2017 Apr 20;17:283. doi: 10.1186/s12885-017-3260-2 (PMC5399416; doi:10.1186/s12885-017-3260-2)
Supplement: Additional file 1: — Supplementary figure and tables. Disease free survival differences by TNM sub-stage, psychiatric illness by age group. (DOC 109 kb) [file 12885_2017_3260_MOESM1_ESM.doc]

**
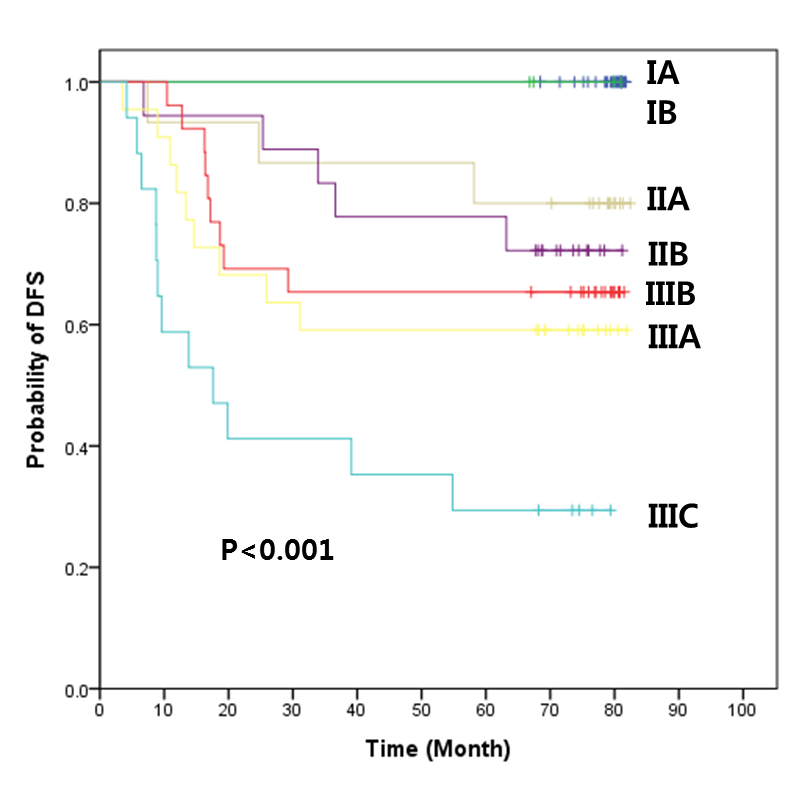
**

**FigureS1. Disease free survival by TNM sub-stage**

Table S1. Psychiatric illness by age group

| Psychiatric illness | Age <60 | Age ≥60 |
| --- | --- | --- |
|  | n=15 (%) | n=12 (%) |
| Adjustment disorder | 11 (73) | 6 (60) |
| Depression | 2 (13) | 3 (25) |
| Anxiety disorder | 2 (13) | 1 (8) |
| Insomnia | 0 | 2 (17) |

Table S2. Odds ratios for psychological distress by patient characteristics

|  |  | | |
| --- | --- | --- | --- |
| Variables | OR | 95% CI | p-value |
| Age | 1.000 | 0.972-1.028 | .991 |
| Gender^a^ | 1.646 | 0.777-3.488 | .193 |
| Performance status^b^ | 1.097 | 0.595-2.020 | .767 |
| Alcohol History^c^ | 1.323 | 0.643-2.723 | .447 |
| Smoking History^d^ | 2.856 | 1.144-7.131 | .025 |
| Education level^e^ | 2.394 | 1.108-5.172 | .026 |
| Marital status^f^ | 1.561 | 0.675-3.611 | .298 |
| Employment status^g^ | 1.203 | 0.593-2.440 | .608 |
| Stage^h^ | 2.716 | 1.466-5.032 | .001 |

a: Male coded 0, Female coded 1

b: ECOG 0 coded 0, ECOG 1-3 coded 1

c: Alcohol Hx No coded 0, Yes coded 1

d: Smoking Hx Yes coded 0, No coded 1

e: >High school coded 0, <Middle school coded 1

f: Married coded 0, Unmarried coded 1

g: Employed coded 0, Unemployed coded 1

h: Stage I-III coded 0, Stage IV coded 1

OR, odds ratio; CI, confidence interval
